# Nano-scale Biophysical and Structural Investigations on Intact and Neuropathic Nerve Fibers by Simultaneous Combination of Atomic Force and Confocal Microscopy

**DOI:** 10.3389/fnmol.2017.00277

**Published:** 2017-08-30

**Authors:** Gonzalo Rosso, Ivan Liashkovich, Peter Young, Victor Shahin

**Affiliations:** ^1^Institute of Physiology II, WWU Münster Münster, Germany; ^2^Department of Sleep Medicine and Neuromuscular Disorders Münster, Germany

**Keywords:** peripheral nervous system, atomic force microscopy, confocal microscopy, biomechanics, biomedical research

## Abstract

The links between neuropathies of the peripheral nervous system (PNS), including Charcot-Marie-Tooth1A and hereditary neuropathy with liability to pressure palsies, and impaired biomechanical and structural integrity of PNS nerves remain poorly understood despite the medical urgency. Here, we present a protocol describing simultaneous structural and biomechanical integrity investigations on isolated nerve fibers, the building blocks of nerves. Nerve fibers are prepared from nerves harvested from wild-type and exemplary PNS neuropathy mouse models. The basic principle of the designed experimental approach is based on the simultaneous combination of atomic force microscopy (AFM) and confocal microscopy. AFM is used to visualize the surface structure of nerve fibers at nano-scale resolution. The simultaneous combination of AFM and confocal microscopy is used to perform biomechanical, structural, and functional integrity measurements at nano- to micro-scale. Isolation of sciatic nerves and subsequent teasing of nerve fibers take ~45 min. Teased fibers can be maintained at 37°C in a culture medium and kept viable for up to 6 h allowing considerable time for all measurements which require 3–4 h. The approach is designed to be widely applicable for nerve fibers from mice of any PNS neuropathy. It can be extended to human nerve biopsies.

## Introduction

Schwann cells (SCs) are of crucial importance for the development, maintenance, and regeneration of the peripheral nervous system (PNS) (Jessen and Mirsky, 2005). They form myelin around peripheral axons, thereby enabling the high-speed propagation of action potentials. In addition to myelin secretion around large and small caliber axons, myelinating SCs, and their associated axons are enwrapped in a continuous basal lamina secreted by SCs which is only 25 nm thick (Thomas, 1963). Different roles have been proposed for this basal lamina, including SC proliferation, survival, migration, and myelination (Colognato et al., 2005; Court et al., 2006; Chernousov et al., 2008; Colognato and Tzvetanova, 2011), but very little is known about its role in the biomechanical support to the nerve fiber. Interestingly, it has been suggested that impaired peripheral nerve biomechanics is directly linked to various neuropathies (Suter and Scherer, 2003; Bai et al., 2010), but the links between nerve biomechanics and neuropathies remain poorly understood. Only a few experiments have described the biomechanical properties of peripheral nerves *in vivo* (Driscoll et al., 2002; Topp and Boyd, 2012). In some cases, the mechanical measurements were performed on single nerve fibers (Urbanski et al., 2016). However, the fibers were fixed and therefore the physiological interpretation remains debatable (Heredia et al., 2007; Huang et al., 2011; Rosso et al., 2012). To gain a better understanding of the biomechanics of PNS nerves, we developed a novel strategy enabling biomechanical investigation of isolated living native peripheral nerve fibers based on simultaneous combination of AFM and confocal microscopy. Biomechanical, structural, and functional investigations were carried out on isolated nerve fibers from wild-type animals and the neuropathic animal model *Pmp22*^−/−^. This knockout model is especially relevant because the peripheral myelin protein 22 (PMP22) is closely connected with several hereditary human PNS neuropathies (Suter and Scherer, 2003; Fledrich et al., 2014), including Charcot-Marie-Tooth1A (CMT1A) and hereditary neuropathy with liability to pressure palsies (HNPP). CMT1A and HNPP are associated with mechanical vulnerability and structural remodeling of nerves, in particular myelin and basal lamina of nerve fibers (Amici, 2006; Guo et al., 2014). They are also associated with an increase in the permeability of myelin and disruption of cell junction complexes in the peripheral nerve (Guo et al., 2014). Impaired biomechanical integrity (vulnerability to mechanical compression and lack of recovery) and leakiness are paralleled by severely reduced nerve conduction velocity and are therefore assumed to account for the clinical symptoms (Bai et al., 2010; Guo et al., 2014). The AFM-confocal microscopy setup we present here provides novel insights into understanding the links between nerve fibers biomechanics and neuropathies.

## Materials

### Reagents

#### Nerve isolation and teasing of nerve fibers

- Isoflurane (Forene®, cat. No 8506 Abbott, Germany).
- Forceps (cat. No 91100-16, Fine Science Tools GmbH, Germany).
- Scissors (cat. No 91402-14 and cat. No 91460-11, Fine Science Tools GmbH, Germany).
- Fine microdissection scissors (cat. No 91500-09, Fine Science Tools GmbH, Germany).
- Dumont extra fine tip #5 forceps (cat. No 11254-20, Fine Science Tools GmbH, Germany). Note: Forceps with extra sharp tips improve the mechanical separation of nerve fibers and teasing.
- Microscope round glass coverslips 25 mm round (cat. No 41001125 Assistant, Germany).
- Glass-bottom 35 mm (Wilko wells) dishes (cat. No HBST-3522 The Netherlands)—Note: glass-bottom dishes can be used for nerve fiber teasing instead of glass coverslips.
- 35 mm cell culture plastic petri dishes (cat. No BD Falcon™).
- Cell-Tak™ tissue adhesive (Cornig™ cat. No CB-40240); stored at 4°C.
- Neurobasal medium (Gibco, cat. No 21103-049); stored at 4°C.

#### Confocal imaging and biomechanical measurements

- Fluoromyelin™ Red (cat. No F34652 Invitrogen, CA, USA); stored at 4°C.
- Fluorescein isothiocyanate–dextran 70 kDa (FITC-dextran cat. No 46945 Sigma, USA).
- V-shaped MSCT silicon nitride AFM cantilevers (Bruker, USA).

#### Viability test of isolated nerve fibers (see Supplementary Material)

- 5-Bromouridine 98%, BrU (cat. No 850187 Sigma, USA).
- Monoclonal Anti-BrdU antibody (cat. No B8434 Sigma, USA).
- Phosphate Buffered Saline (PBS) (cat. No L 1825, Biochrom, Germany).
- Normal Goat Serum (cat. No 31872 Invitrogen, CA, USA).
- Paraformaldehyde (cat. No 0335.2 Roth, Germany).

#### Topography investigations of intact and basal lamina–disrupted nerve fibers (see Supplementary Material)

- CLSPA collagenase enzyme (cat. No. LS005275 Worthington, USA); stored at 2–8°C.
- V-shaped MSCT silicon nitride cantilevers (Bruker, USA).

### Equipment

- Binocular stereo microscope (Stemi 305, Zeiss).
- Atomic Force Microscope (Nanowizard® 3 AFM system, JPK, Germany).
- Laser scanning confocal microscope Leica TCS SP8 (DMI6000) equipped with Leica Application Suite Advanced Fluorescence (LAS AF) software (Leica, Germany).
- Imaging chamber with controlled temperature BioCell^TM^ or petri-dish heater for Nanowizard® 3 AFM (JPK instruments, Germany).
- Laboratory incubator (Model BD115, Binder GmbH, Germany).
- 10X air and 63X oil NA 1.40 objectives (Leica, Germany).
- Immersion oil Type F, *n* = 1.518 (Leica, Germany).
- Data analysis and image processing software: Origin 2016 (OriginLab Corporation, USA).

### Experimental design

AFM is simultaneously combined with confocal microscopy to investigate native never fibers from multiple structural and biophysical aspects. AFM is applied as a mechanical and structural nano-tool alike. It enables structural investigation of nerve fibers surface at nano-resolution. At the same time, it is used to indent nerve fibers and study their response to the mechanical load. The simultaneous combination with confocal microscopy is used to perform biomechanical, structural and functional integrity measurements at nano- to micro-scale.

### Application and target audience

The role of biomechanics in diverse biomedical research areas is gaining substantial attention. This includes development, maintenance, differentiation of cells, and tissues as well as diseases (Ingber, 2003; Engler et al., 2006; Wang et al., 2009; Franze et al., 2013). The protocol presented here combines the biophysical nano-approach AFM and confocal microscopy simultaneously to investigate biomechanics of native peripheral nerve fibers from wild-type and neuropathic mice. Hence, this step-by-step protocol can be applied to investigate biomechanical properties of a variety of mouse animal models of peripheral neuropathies such as CMT1A among others (Sereda et al., 1996). In addition, this protocol can be utilized to investigate nerve biomechanics of young animals including rats, extending its applicability to an increased number of PNS diseases (Meyer Zu Hörste and Nave, 2006). Nerves may be investigated in the same way as their individual building blocks. Nerve biopsies from human patients with PNS neuropathies can also be tested. Such investigations will provide valuable information about the biomechanical changes in the peripheral nervous tissue during development and disease. All in all, the protocol targets a broad audience from biophysical, biomedical and clinical research.

### Step-by-step protocol (timing for each step)

The steps in sections 4 and 5a involve the preparation of the AFM-Confocal system for biomechanical and viability investigations. These can be done before step 1 in order to save time (~30 min) between the sample preparation and the measurements.

**Figure 1:**
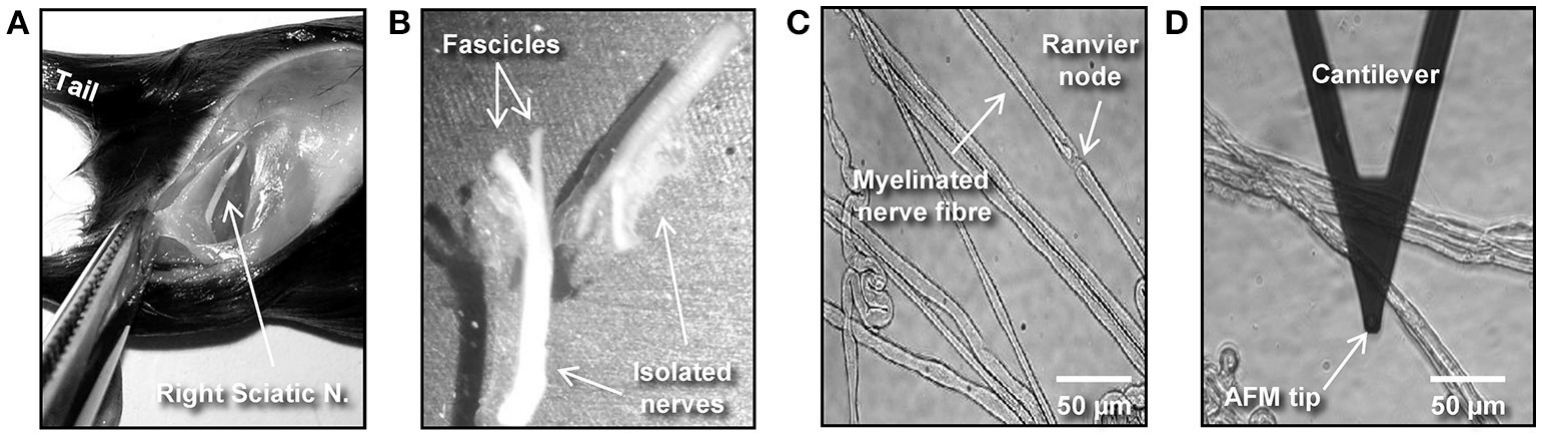
Isolation and preparation of mouse peripheral nerve fibers for biomechanical investigations. **(A)** The image shows a hind-limb of a C57BL/6 mouse where the skin and muscles were cut open to expose the right-side sciatic nerve. **(B)** Dissected left and right sciatic nerves after mechanical removal of the epineurium. **(C)** Phase contrast image showing individual (teased) myelinated nerve fibers spread over a previously Cell-Tak®–coated glass surface. **(D)** Phase contrast image showing an AFM cantilever in the proximity of a myelinated nerve.

**Figure 2:**
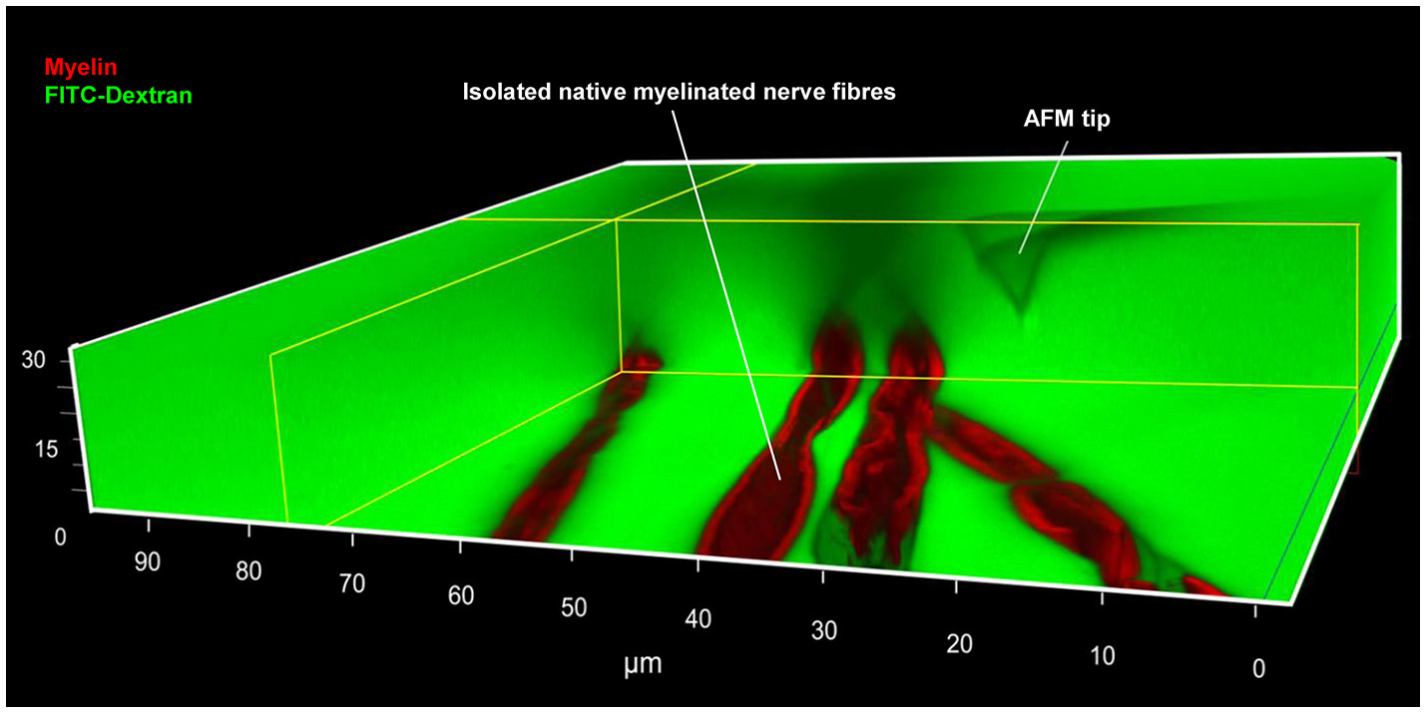
Simultaneous AFM-Confocal microscopy measurements for biomechanical and integrity investigations on nerve fibers. A 3D image showing the combination of AFM and confocal microscopy to study myelinated nerve fibers harvested from sciatic nerves of adult mice. Isolated nerve fibers are maintained at 37°C in a culture medium containing fluorescent myelin marker (red) and 70 kDa FITC-dextran (green). FITC-dextran fulfills two roles: First, the non-fluorescent and unlabeled AFM tip becomes visible as a negative image. The AFM tip can then be moved precisely to an area a nerve fiber area of interest (within nodes and internodes). Second, intact nerve fibers are impermeable to 70 kDa FITC-dextran owing to their functional integrity. Loss of integrity (biomechanical, structural, or functional) will lead to FITC-dextran leakiness into the interior of the fibers. A rectangular section containing several confocal planes is digitally removed from the image to enable the visualization of the non-fluorescent AFM tip as well as the non-labeled axon (seen as a longitudinal semi-tube). Modified from Rosso et al. (2014).

**Figure 3:**
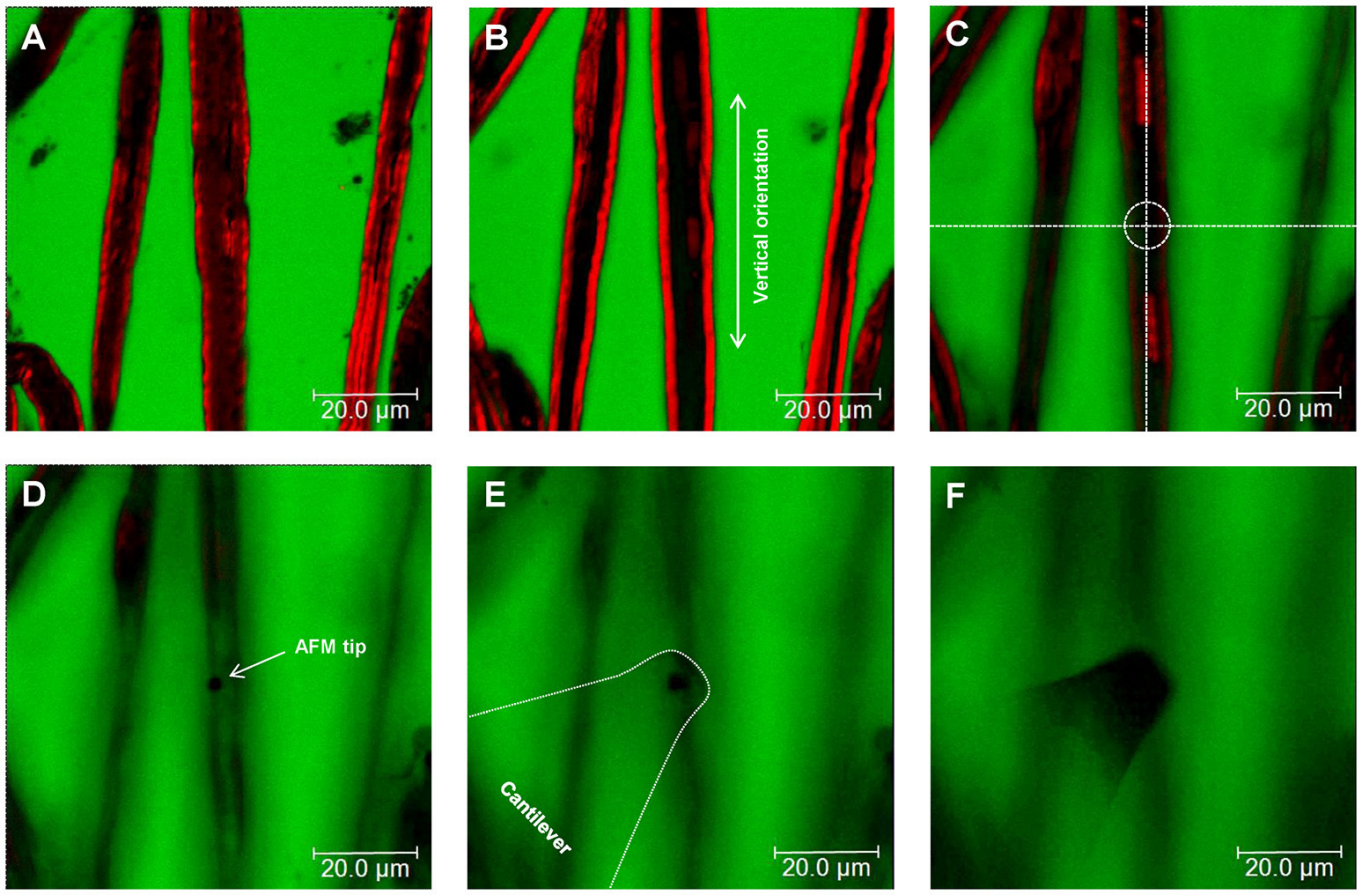
Nerve fiber orientation and AFM tip positioning for AFM-Confocal microscopy based biomechanical investigations. **(A–F)** Confocal images showing consecutive z-stacks (0.5 μm) of teased myelin-labeled nerve fibers (red) immersed in FITC-dextran (green) from bottom **(A)** to top **(F)**. **(C–F)** Positioning of the AFM tip to an area of interest (dotted circle in **C**) on the surface of a selected nerve fiber.

**Figure 4:**
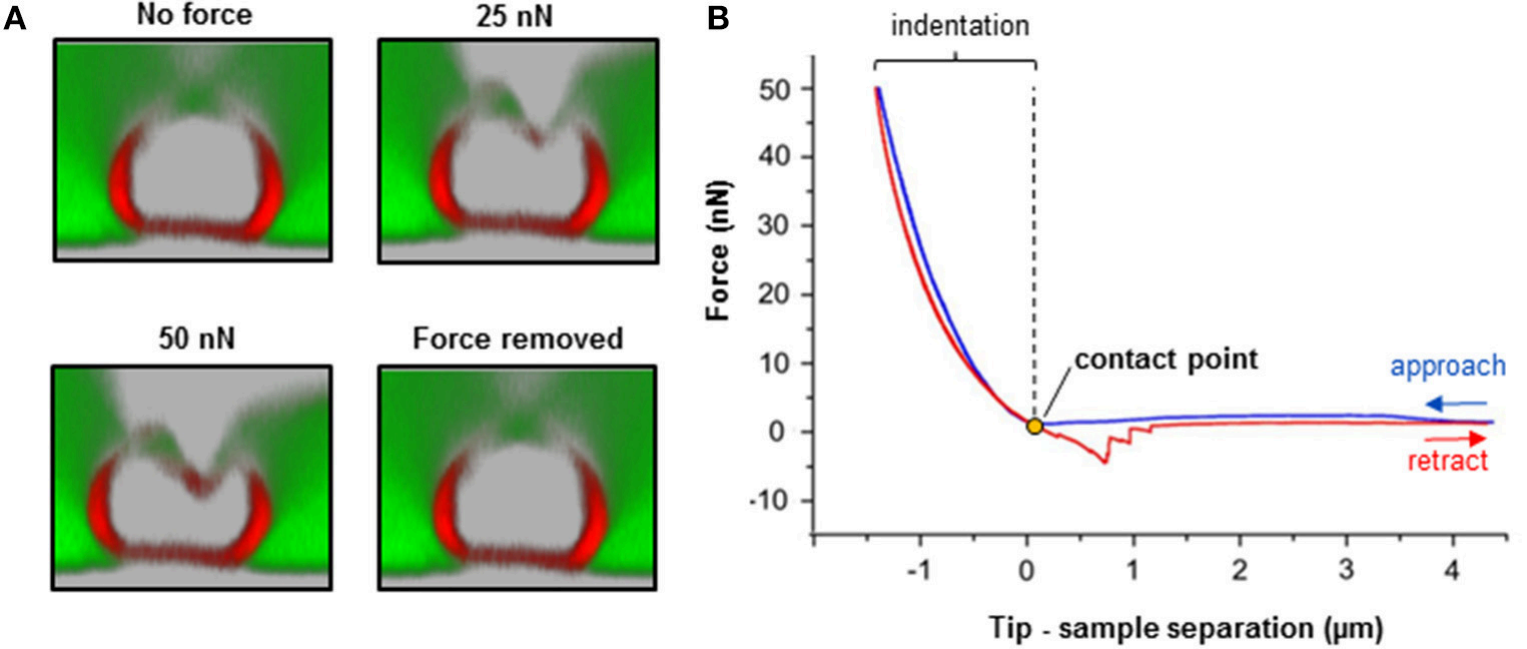
Biomechanical integrity investigations on isolated nerve fibers. **(A)** Simultaneous combination of AFM and confocal microscopy for biomechanical measurements on an individual myelinated nerve fiber. Transversal confocal section images visualizing the response of a nerve fiber to mechanical indentation upon exposure to incremental loading forces (25 and 50 nN), and after force removal. Scale bar in A = 5 μm. **(B)** The AFM approaches the sample to start the indentation (blue curve). Then, it retracts and breaks loose of the sample (red curve) in order to start a new approach-retract cycle. The recorded force-separation or force-deformation curve can be used to obtain numerous parameters; for instance, indentation depth, stiffness (slope of the fitted approach curve) and elastic modulus (using the appropriate model, which depends on the shape of the AFM tip as an indenter; refer to “Point 7: Data interpretation and analysis ”) (Rosso et al., 2014). Loss of mechanical integrity may be detectable as ruptures or “breakthrough” events in the curve (Rosso et al., 2014). Modified from Rosso et al. (2014).

**Figure 5:**
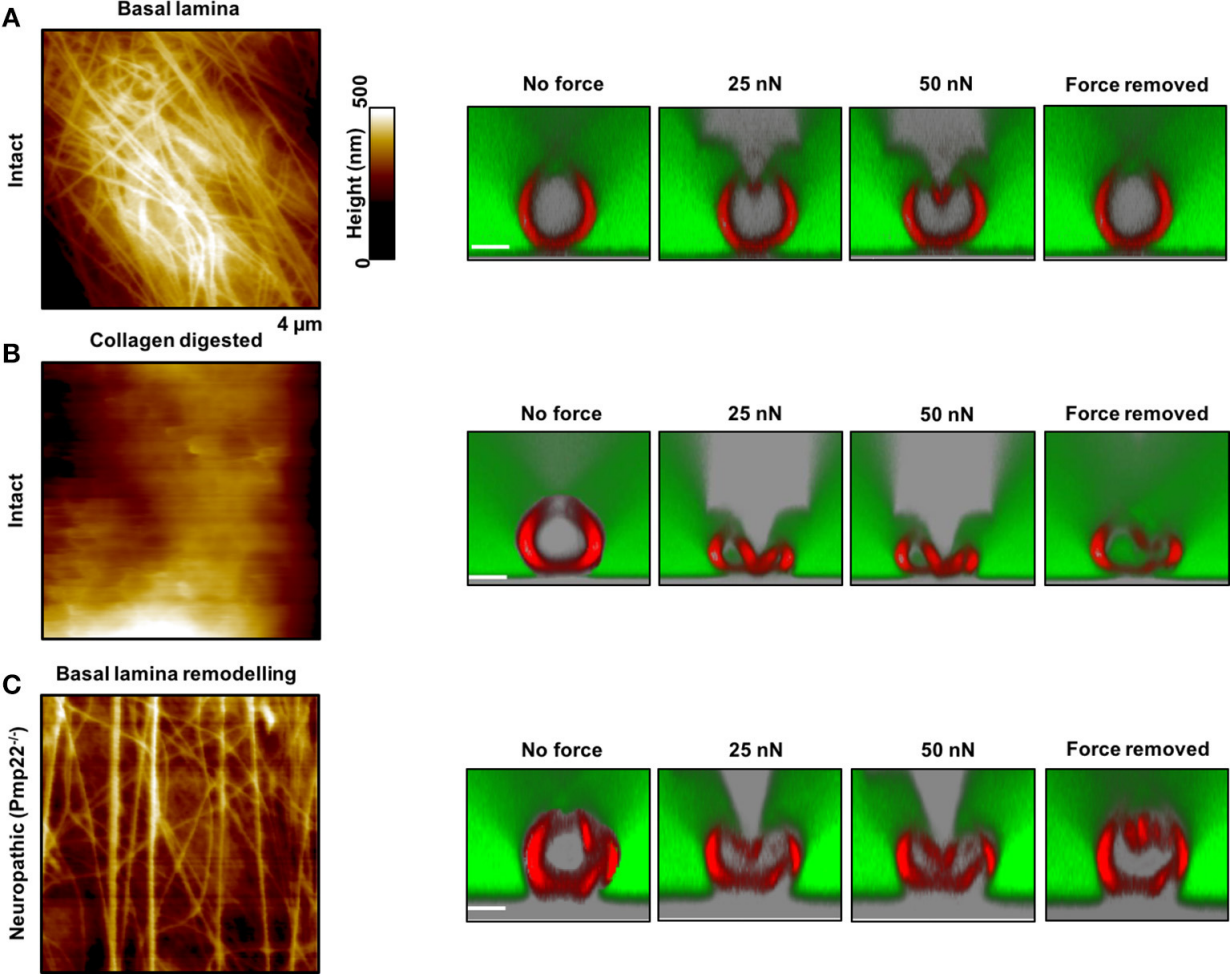
Contribution of the basal lamina and PMP22 to biomechanical and structural integrity of myelinated peripheral nerve fibers. **(A,B)** Left: AFM images of control and basal lamina collagen–stripped nerve fibers, respectively, from intact mice. Right: AFM-based compression of intact nerve fibers to study and visualize simultaneously their response to mechanical forces and to force removal. The compressing AFM-tip is seen in gray in the images on the right. Obviously, the basal lamina provides nerve fibers with remarkable biomechanical integrity and enables them to recover promptly from substantial mechanical compression. Collagen removal from the basal lamina results in loss of mechano-protection and in nerve fibers leakiness to 70 kDa FITC-dextran (green). **(C)** Left: AFM image of the basal lamina of nerve fibers from neuropathic mice (*Pmp22*^−/−^). Right: Response of *Pmp22*^−/−^ nerve fibers to mechanical compression. Absence of PMP22 is paralleled by structural remodeling of the basal lamina and severe mechanical vulnerability. Scale bars in confocal images are 5 μm each. Modified from Rosso et al. (2014).

1. Preparation of Adhesive Surfaces (Time: 10 min)
  Nerve fibers can be mechanically separated (teased) on 25 mm glass coverslips or 35 mm glass-bottom dishes coated with the adhesive protein CellTak™.
  - For coverslips: Clean 25 mm round glass coverslips by first immersing them in 70% ethanol and then in dH_2_O. Repeat four times both the ethanol and dH_2_O washes. Start and finish with 70% ethanol. Let surfaces dry out (2 min).
  - For coverslips and glass-bottom dishes: Cover the glass surface with tissue adhesive Cell-Tak™ (mixed in + 5% acetic acid) and mechanically spread the liquid over the surface with a pipette tip to form a thin layer. After the acetic acid evaporates, a film of Cell-Tak™is left behind.
  - Wash the surface two times with 70% ethanol and two times with dH_2_O by pipetting up and down. Let the surface dry out for 5 min. Note: Prepared substrates can be stored at 4°C for several days. Troubleshooting: we recommend immediate use for maximum adhesion of nerve fibers to the surface.
2. Isolation of Mouse Sciatic Nerve AND Teasing of Nerve Fibers (Time: 25–30 min) This part of the protocol describes a step-by-step procedure to isolate adult mouse peripheral nerve fibers for biomechanical AFM-Confocal indentation measurements. Note: The protocol can be adjusted to young, wild-type, and different neuropathic animal models.
  - Anesthetize the animal using an overdose of isoflurane contained in a closed receptacle under fume hood. Wait ~1 min for the animal to fall asleep. Remove the anesthetized mouse from the container and kill the animal by applying cervical dislocation—in accordance with the European Convention for Animal Care and Ethical Use of Laboratory Animals (State Office for Nature, Environment and Consumer Protection, North Rhine-Westphalia, Germany; File reference 84-02.05.20.12.146).
  - Spray 70% ethanol over the mouse's hind-limbs to dampen the fur. Using scissors make an incision at the level of mid-thigh (Figure 1A) to expose the leg muscles. Introduce the scissor between the muscle junction to separate them and expose the sciatic nerve.
  - Using forceps, thoroughly separate the sciatic nerve from the muscles and using microdissection scissors, cut a 1 cm segment. Repeat the same procedure to dissect the sciatic nerve on the opposite leg. Troubleshooting: special care must be taken to avoid stretching the nerve during dissection. The latter can cause changes in myelin structure and nerve fiber degeneration.
  - Transfer the sciatic nerves to a 35 mm plastic petri dish halfway filled with ice-cold Neurobasal medium.
  - The following steps are performed under binocular stereomicroscope. Remove (peel off) the perineurial tissue using two extra fine tip #5 forceps. This can be performed by holding one end of the nerve with one forceps and thoroughly stripping away the perineurium with the second forceps. After perinuerium removal, different caliber fascicles are visible (Figure 1B). Note: This step is crucial to release the nerve fascicles from the ensheathing connective tissue and facilitates the fibers' mechanical separation.
  - Using the same set of extra fine tip forceps, separate individual fascicles from the main nerve trunk and transfer one edge of a Cell-Tak™-pre-coated glass surface. Add a drop of ice-cold Neurobasal medium over the transferred fascicle if necessary to avoid drying out.
  - Holding the end of a bundle of nerve fibers with forceps, separate a small number of the fibers from the main bundle. Using the same sharp tip forceps (alternatively a very sharp needle), further separate the nerve fibers from each other very gently by pushing them across the pre-coated surface to areas where Cell-Tak® is still dry. This procedure helps to separate the nerve fibers that promptly adhere to the substrate. Hence, single and separated nerve fibers (Figure 1C) are necessary for biomechanical investigations with the AFM tip (Figure 1D). Potential pitfall: Teasing of nerve fibers is the most challenging step and requires practice and patience.
3. Fluorescent Myelin Labeling (Time: 25 min)
  - Utilizing a pipette, slowly add small drops of 37°C heated Neurobasal pre-staining medium (containing 5 μl/ml of Fluoromyelin™ Red) until nerve fibers are completely covered (~0.8 ml/coverslip and 1 ml for 35 mm glass-bottom dishes). Flouromyelin^TM^ is a fluorescent dye that selectively stains for myelin sheaths (Monsma and Brown, 2012). Troubleshooting: Turbulence generated by vigorous pipetting may cause the fiber to detach from the adhesive surface. Tip: Slowly and smoothly apply the myelin pre-staining medium while the pipette tip is in contact with the glass surface.
  - Incubate the samples in an incubator at 37°C for 20 min. Check samples every 5 min to make sure the fibers do not dry out. Add medium if necessary.
4. AFM Setup Calibration (Time: 20 min) Points (a–e) in this section involve the calibration of the AFM tips. We recommend that this is performed before steps 1 and 2.
  - Place a clean 25 mm glass coverslip or a 35 mm glass-bottom petri dish onto the AFM stage. Add pre-warmed 37°C Neurobasal medium to the imaging chamber. Set the temperature of the image chamber to 37°C at least 15 min before starting the calibration to allow temperature stabilization.
  - Mount the MSCT probe onto the AFM-Confocal setup and calibrate the cantilever spring constant by first calculating the deflection sensitivity.
  - After adjusting the AFM-laser position onto the back side of the cantilever and the vertical and horizontal voltage to zero in the photodiode, drive down the AFM tip to reach a tip-sample distance of ~4–5 μm. Push the tip against a clean uncoated glass surface. Record a number of force-distance curves.
  - Calculate the deflection sensitivity values by utilizing the portion of the curve that represents the physical contact between the tip and the glass surface.
  - Retract the AFM tip from the surface (~100 μm) and calculate the spring constant in liquid using the thermal noise method (te Riet et al., 2011).
5. Confocal Set Up and Nerve Fiber Imaging (Time: 10 min)
  - Turn on all the electronic equipment associated with the confocal microscope including the 488 and 633 lasers lines. Set the microscope objective at 10X.
  - Remove the AFM head from the confocal microscope stage.
  - Take the teased nerve fibers immersed in myelin pre-straining medium from the incubator and place them in the microscope's imaging chamber. Keep the temperature constant at 37°C throughout the experiment.
  - Replace the pre-staining solution with pre-warmed 37°C Neurobasal medium containing 1 mg/ml of 70 kDa FITC-dextran.
  - Bring back the AFM head to the microscope's stage. Potential pitfall: Special care must be taken to keep the position of the AFM laser unchanged throughout the remaining steps. Troubleshooting: If the laser position changes, recalibrate the cantilever's deflection sensitivity and the spring constant (step 4).
  - Manually place the AFM tip on the center of the image field using knobs or the AFM stage controller. Switch to 63X immersion oil objective.
  - Image the nerve fibers with the confocal microscope at 1024 x 1024 pixels (approximate image size 100 × 100 μm) until a region is found where the nerve fibers are sufficiently separated from one another, and whereby the nerve fiber in view shows homogeneous myelin staining and structural integrity (Figure 2). Potential pitfall: Breaks or damage on the nerve fibers that occurred during mechanical separation (teasing) increase the presence of FITC-Dextran in the fibers' interiors and can be visualized with the confocal microscope (Supplementary Figure 1).
  - Once a nerve fiber is targeted, drive the AFM tip down to a distance of 25–30 μm from the glass surface.
  - Change the (X,Y) coordinates on the AFM software in order to position the AFM tip close to an individual nerve fiber (Figure 3 and Supplementary Video 1).
6. Simultaneous AFM-Confocal Measurements (Time: 3–4 h)
  - Once the tip is placed above a myelinated axon, rotate the confocal scanning angle in order to orient the nerve fiber vertically (Figure 3B). Note: Vertical orientation of nerve fibers is important to generate transverse views of myelinated axons (Figure 4).
  - For complete visualization of the nerve fiber and the AFM tip (XYZ images) using a confocal microscope, it is recommended to generate 60 z-stacks of 0.5 μm spaces for the two confocal channels. Note: Larger spaces between z-stacks can be chosen in order to speed up the image acquisition. Potential pitfall: The increase in space between z-stacks generates confocal images with poor vertical resolution.
  - Select a resolution of 1,024 × 16 pixels in order to image a small cylindrical section (XYZ confocal image) of the myelinated axon. Make sure that the AFM tip is placed in the center of the image and above the nerve fiber as shown in Figure 3C. Note: The best confocal (XYZ) images of transverse nerve sections are generated when the fibers are vertically oriented and the AFM tip is positioned above the nerve fibers in the center of the field (Supplementary Video 1).
  - Run a complete confocal image without applying force to the nerve fiber at 1,024 × 16 pixels (Figure 4A, No force) and register the time interval (in seconds) necessary for the microscope to complete the XYZ image (it takes ~15–20 s for large-caliber fibers).
  - Using the LAS Montage 3D viewer tool, rotate the image 90° and verify the quality of the image (see examples in Figures 4, 5).
  - Use the time interval calculated (in point d) and set it as the z-piezo extension delay (tip-sample delay). Note: This is the time that the AFM tip remains indenting the sample before retraction. For large-caliber fibers, set tip-sample delay to around 20 s.
  - Set the tip-nerve fiber distance to ~4–5 μm and the tip velocity to 1 μm s^−1^ in closed z-loop. Run a force-distance curve at a given loading force. Simultaneously scan the nerve fiber with the confocal microscope. Troubleshooting: The time necessary for a complete z-piezo extension-retraction cycle should be long enough to allow the confocal microscope to complete the z-stack scanning of the whole nerve fiber and the AFM tip.
  - Apply different loading forces (e.g., 25 and 50 nN) to image with the confocal any variations in nerve fiber deformation during compression (Figure 5).
  - For data collection and statistical analysis, probe the elasticity of the nerve fiber with the AFM tip and record ~5 force-indentation curves at the same position (Figure 4B). Note: In this case use zero (0) seconds for the tip-sample extension and retraction delay.
7. Data Interpretation and Analysis
  - Elastic Young's modulus determination in nerve fibers. Open the images with the AFM data analysis. Convert the obtained force-distance curves to force-indentation curves by correcting the cantilever deflection caused by the piezo movement. Provide the software (if necessary) with the calculated cantilever's spring constant. The elastic Young's modulus of nerve fibers is determined with the Hertz-Sneddon model corrected for pyramidal indenters (Rico et al., 2005). For a sharp pyramidal AFM tip, the tip-sample force (F) is proportional to the square of the sample indentation (δ), and its relation is given by the following equation:
    $$\begin{array}{l} F=\frac{1}{\sqrt{2}}\frac{Etan\alpha }{\left(1-v^{2}\right)}\delta^{2} \end{array}$$
    *F* is the loading force, E is the elastic Young's modulus, ν is the Poisson's ratio of the sample (ν = 0.5 is a value commonly used for cells), α is the half-opening angle to the face of the tip (α = 10.7°) and δ is the indentation depth. Note: For the calculation of the MSCT tip semi-included face angle, a regular pyramid with a 15° vertex angle (based on the manufacturer information) can be assumed. The corresponding semi-included angle to the face is ~10.7°.
  - Force-curve fitting and biomechanical analysis. Elasticity values from wild-type and neuropathic nerve fibers can be obtained by fitting the slope of the recorded force-indentation curves using software analysis. For the calculation of overall nerve fiber elasticity (Young's modulus) only the extension curve (approach) is considered. The elasticity is determined by compressing the nerve fiber at a given loading force. The resulting force-indentation curve can be fit from the contact point to the end (maximum indentation, Figure 4B). Curve fitting analysis can be carried out using different computer programs such as Protein Unfolding and Nano-Indentation Software (PUNIAS, site.voila.fr/punias) or JPK/SPM data processing software (JPK, Germany, www.jpk.com) among others. Estimated elasticity values can be exported and processed using Origin Pro 8.0 software for graphic production and statistical analysis (Origin Lab Corporation, USA).

## Advantages and limitations of the experimental approach

### Advantages

- The method has broad applicability to investigate biophysical properties of biological samples at the nano-and macro-scale levels.
- Samples are preserved in their native state without any chemical treatment throughout the whole process of investigation.
- Biomechanical (AFM) and structural (confocal) measurements are carried out simultaneously and correlated immediately and directly to one another. The vast majority of related publications so far have investigated the biomechanical and structural integrity of biological samples separately from one another.
- The method is adaptable to the demands of the samples of interest (normal and pathological nerve samples, such as demyelinopathies and axonopathies).

### Disadvantages

- In-depth training is needed to perform the teasing of nerve fibers and safely use the combined AFM-Confocal setup.
- Biomechanical investigations are limited to samples that are not too stiff for AFM tips.
- Measurements can only be done *in vitro*.

## Anticipated results

Neuropathic animal models, *Pmp22*^−/−^, exhibit biomechanical vulnerability and are prone to conduction block when exposed to compression (Bai et al., 2010). We anticipate significant differences in biomechanical, structural and functional integrity between intact and neuropathic nerve fibers. Representative AFM-Confocal images for biomechanical, functional, and structural integrity investigations on myelinated nerve fibers from intact and *Pmp22*^−/−^ mice are shown in Figures 4, 5. Nerve fibers are kept in a medium containing 70 kDa dextran. They are then exposed to incremental loading forces and their response is tested. The high molecular weight dextran is excluded from axonal entry unless fibers lose their biomechanical, structural or functional integrity.

### Surface morphology

The basal lamina of nerve fibers from *Pmp22*^−/−^ mice is assumed to be loose (Amici, 2006) and we therefore anticipate marked differences in the surface architecture of *Pmp22*^−/−^ compared to intact fibers. As a matter of fact, *Pmp22*^−/−^ nerve fibers exhibit a basal lamina arrangement pattern (parallel) which is significantly different from the pattern in intact fibers (interwoven) (Figure 5). The basal lamina is crucial for mechanoprotection of nerve fibers. Hence, we expect that alteration in its structure will impair the biomechanical integrity of nerve fibers. Indeed, chemical digestion of collagen from the basal lamina is paralleled by high vulnerability to mechanical compression (Figure 5).

### Elasticity/viscoelasticity

Fully elastic materials strain when stretched and rapidly restore their original shape once the stress is removed. Viscoelastic materials possess both elastic and viscosity properties and exhibit time-dependent strain rate. The elastic Young's modulus provides information about stiffness and elasticity and can be derived from force-deformation curves obtained with AFM (Figure 4 and “Point 7: Data interpretation and analysis”). Likewise, the elastic/viscoelastic behavior of fibers can be derived from the force-deformation curves upon fibers indentation (Figure 4).

We anticipate no full elastic behavior of nerve fibers. We also anticipate significantly lower elastic modulus of *Pmp22*^−/−^ compared to intact fibers. Indeed, the apparent overall elasticity of myelinated nerve fibers from intact mice (31.4 ± 8.8 kPa, *N* = 11) is significantly higher compared to *Pmp22*^−/−^ (15.6 ± 5.2 kPa, *N* = 11) fibers (Rosso et al., 2014). Viscoelasticity presents as an hysteresis loop in force-deformation curves; approach and retract curves are non-linear and do not coincide. Intact nerve fibers show viscoelastic behavior whereas (Figure 4) *Pmp22*^−/−^ are plastic and thus strain irreversibly when exposed to stress (Rosso et al., 2014).

### Recovery time

This is the average time necessary for nerve fibers to recover after force removal. We expect either a lack or slowdown of recovery time after compression in neuropathic nerve fibers. Based on the analysis of force-indentation curves, it takes intact nerve fibers 2–6 s to fully recover after removal of the force (Figure 5A, right image). In contrast, nerve fibers from *Pmp22*^−/−^ mice fail to recover (Figure 5C). Digestion of collagen from the basal lamina of intact fibers deprives them of the ability to recover (Figure 5C).

### Functional, structural, and biomechanical integrity

Functional, structural, and biomechanical integrity is crucial for high conduction velocity of myelinated nerve function. We therefore anticipate mechanical resilience of intact myelinated nerve fibers. As a matter of fact, exposure of intact myelinated nerve fibers to substantial mechanical forces in incremental steps to the extent of squeezing fail to inflict structural or functional damage upon them. The sharp and stiff AFM tip can be likened to a needle exerting remarkable local pressure while mechanically probing the fibers, and yet the resulting pressure is resisted. Not only the axon caliber but also the tightness of myelin which is reinforced by the correct assembly of compact myelin proteins such as PMP22 is critical to ensure the high conduction velocity (Bai et al., 2010). Increase in nerve fiber permeability across the myelin sheath in paranodal regions has been shown to severely reduce the nerve conduction velocity (Guo et al., 2014). Therefore, structural, functional, and biomechanical integrity of intact nerve fibers naturally prevents leakiness. We anticipate leakiness to naturally excluded large macromolecules, 70 kDa FITC-dextran, in nerve fibers lacking biomechanical integrity. Consistently, following digestion of collagens from the mechanoprotective basal lamina in nerve fibers from intact mice, leakiness is visualized as dextran entry into axons in combined AFM-Confocal images (Rosso et al., 2014).

### Breakthrough events

Lack of mechanical resilience is detectable as “breakthrough events” in force-deformation curves. These events represent the disruption, softening and destabilization of nerve fibers when mechanically probed by the sharp AFM tip during sample indentation. They can be visualized in the approach curves as “jagged zones.” The zones are an indicator of nerve fiber vulnerability to compression or impaired mechanical resilience (Rosso et al., 2014).

## Author contributions

VS and GR designed the experiments, analyzed data, and wrote up the manuscript paper. GR carried out all the experiments, analyzed data, and prepared the figures. PY and IL contributed to the design of the experiments and the paper, and were actively involved in the analysis of the data and provided essential inputs.

### Conflict of interest statement

The authors declare that the research was conducted in the absence of any commercial or financial relationships that could be construed as a potential conflict of interest.

## References

[B1] Amici S. A. (2006). Peripheral myelin protein 22 is in complex with 6beta4 integrin, and its absence alters the schwann cell basal lamina. J. Neurosci. 26, 1179–1189. 10.1523/JNEUROSCI.2618-05.2006 16436605 PMC6674566

[B2] Bai Y. Zhang X. Katona I. Saporta M. A. Shy M. E. O'Malley H. A.. (2010). Conduction block in PMP22 deficiency. J. Neurosci. 30, 600–608. 10.1523/JNEUROSCI.4264-09.2010 20071523 PMC3676309

[B3] Chernousov M. A. Yu W.-M. Chen Z.-L. Carey D. J. Strickland S. (2008). Regulation of Schwann cell function by the extracellular matrix. Glia 56, 1498–1507. 10.1002/glia.20740 18803319

[B4] Colognato H. Tzvetanova I. D. (2011). Glia unglued: how signals from the extracellular matrix regulate the development of myelinating glia. Dev. Neurobiol. 71, 924–955. 10.1002/dneu.20966 21834081 PMC5679221

[B5] Colognato H. Ffrench-Constant C. Feltri M. L. (2005). Human diseases reveal novel roles for neural laminins. Trends Neurosci. 28, 480–486. 10.1016/j.tins.2005.07.004 16043237

[B6] Court F. A. Wrabetz L. Feltri M. L. (2006). Basal lamina: schwann cells wrap to the rhythm of space-time. Curr. Opin. Neurobiol. 16, 501–507. 10.1016/j.conb.2006.08.005 16956757

[B7] Driscoll P. J. Glasby M. A. Lawson G. M. (2002). An *in vivo* study of peripheral nerves in continuity: biomechanical and physiological responses to elongation. J. Orthop. Res. 20, 370–375. 10.1016/S0736-0266(01)00104-8 11918319

[B8] Engler A. J. Sen S. Sweeney H. L. Discher D. E. (2006). Matrix elasticity directs stem cell lineage specification. Cell 126, 677–689. 10.1016/j.cell.2006.06.044 16923388

[B9] Fledrich R. Stassart R. M. Klink A. Rasch L. M. Prukop T. Haag L.. (2014). Soluble neuregulin-1 modulates disease pathogenesis in rodent models of Charcot-Marie-Tooth disease 1A. Nat. Med. 20, 1055–1061. 10.1038/nm.3664 25150498

[B10] Franze K. Janmey P. A. Guck J. (2013). Mechanics in neuronal development and repair. Annu. Rev. Biomed. Eng. 15, 227–251. 10.1146/annurev-bioeng-071811-150045 23642242

[B11] Guo J. Wang L. Zhang Y. Wu J. Arpag S. Hu B.. (2014). Abnormal junctions and permeability of myelin in PMP22-deficient nerves. Ann. Neurol. 75, 255–265. 10.1002/ana.24086 24339129 PMC4206215

[B12] Heredia A. Bui C. C. Suter U. Young P. Schäffer T. E. (2007). AFM combines functional and morphological analysis of peripheral myelinated and demyelinated nerve fibers. Neuroimage 37, 1218–1226. 10.1016/j.neuroimage.2007.06.007 17689984

[B13] Huang W.-C. Liao J.-D. Lin C.-C. K. Ju M.-S. (2011). Depth-sensing nano-indentation on a myelinated axon at various stages. Nanotechnology 22:275101. 10.1088/0957-4484/22/27/275101 21597149

[B14] Ingber D. E. (2003). Mechanobiology and diseases of mechanotransduction. Ann. Med. 35, 564–577. 10.1080/07853890310016333 14708967

[B15] Jessen K. R. Mirsky R. (2005). The origin and development of glial cells in peripheral nerves. Nat. Rev. Neurosci. 6, 671–682. 10.1038/nrn1746 16136171

[B16] Meyer Zu Hörste G. Nave K.-A. (2006). Animal models of inherited neuropathies. Curr. Opin. Neurol. 19, 464–473. 10.1097/01.wco.0000245369.44199.27 16969156

[B17] Monsma P. C. Brown A. (2012). FluoroMyelin^TM^ Red is a bright, photostable and non-toxic fluorescent stain for live imaging of myelin. J. Neurosci. Methods 209, 344–350. 10.1016/j.jneumeth.2012.06.015 22743799 PMC3429707

[B18] Rico F. Roca-Cusachs P. Gavara N. Farré R. Rotger M. Navajas D. (2005). Probing mechanical properties of living cells by atomic force microscopy with blunted pyramidal cantilever tips. Phys. Rev. E Stat. Nonlinear Soft Matter Phys. 72, 1–10. 10.1103/PhysRevE.72.021914 16196611

[B19] Rosso G. Liashkovich I. Gess B. Young P. Kun A. Shahin V. (2014). Unravelling crucial biomechanical resilience of myelinated peripheral nerve fibres provided by the Schwann cell basal lamina and PMP22. Sci. Rep. 4:7286. 10.1038/srep07286 25446378 PMC4250911

[B20] Rosso G. Negreira C. Sotelo J. R. Kun A. (2012). Myelinating and demyelinating phenotype of Trembler-J mouse (a model of Charcot-Marie-Tooth human disease) analyzed by atomic force microscopy and confocal microscopy. J. Mol. Recogn. 25, 247–255. 10.1002/jmr.2176 22528185

[B21] Sereda M. Griffiths I. Pühlhofer A. Stewart H. Rossner M. J. Zimmerman F.. (1996). A transgenic rat model of Charcot-Marie-Tooth disease. Neuron 16, 1049–1060. 8630243 10.1016/s0896-6273(00)80128-2

[B22] Suter U. Scherer S. S. (2003). Disease mechanisms in inherited neuropathies. Nat. Rev. Neurosci. 4, 714–726. 10.1038/nrn1196 12951564

[B23] te Riet J. Katan A. J. Rankl C. Stahl S. W. van Buul A. M. Phang I. Y.. (2011). Interlaboratory round robin on cantilever calibration for AFM force spectroscopy. Ultramicroscopy 111, 1659–1669. 10.1016/j.ultramic.2011.09.012 22094372

[B24] Thomas P. K. (1963). The connective tissue of peripheral nerve: an electron microscope study. J. Anat. 97, 35–44. 13981107 PMC1244253

[B25] Topp K. S. Boyd B. S. (2012). Peripheral nerve: from the microscopic functional unit of the axon to the biomechanically loaded macroscopic structure. J. Hand Ther. 25, 142–152. 10.1016/j.jht.2011.09.002 22133662

[B26] Urbanski M. M. Kingsbury L. Moussouros D. Kassim I. Mehjabeen S. Paknejad N.. (2016). Myelinating glia differentiation is regulated by extracellular matrix elasticity. Sci. Rep. 6:33751. 10.1038/srep33751 27646171 PMC5028715

[B27] Wang N. Tytell J. D. Ingber D. E. (2009). Mechanotransduction at a distance: mechanically coupling the extracellular matrix with the nucleus. Nat. Rev. Mol. Cell Biol. 10, 75–82. 10.1038/nrm2594 19197334

