# Supplementary material for: Nano-scale Biophysical and Structural Investigations on Intact and Neuropathic Nerve Fibers by Simultaneous Combination of Atomic Force and Confocal Microscopy
Source: Front Mol Neurosci. 2017 Aug 30;10:277. doi: 10.3389/fnmol.2017.00277 (PMC5582161; doi:10.3389/fnmol.2017.00277)
Supplement: Supplementary file 2 [file DataSheet1.DOCX]

**SUPPLEMENTARY INFORMATION**

*Protocol for viability assay of living mouse isolated peripheral nerve fibers*

This protocol describes an assay to verify the viability of isolated nerve fibers based on their ability to synthesize new RNA (Jao and Salic, 2008; Rosso et al., 2014). The presence of newly-transcribed RNA inside the nerve fibers is investigated at different incubation times by immunofluorescence (Rosso et al., 2014).

1. Immediately after nerve fibre teasing, incubate the samples in Neurobasal medium containing 2.5 mM of the uridine analog (5-Bromooxiuridine, BrU).
2. Incubate the nerve fibers at 37°C and 5% CO_2_. Note: The incorporation of BrU into the nerve fibers can be investigated at different incubation times for up to 4 hours.
3. After incubation, wash the samples thoroughly 10 times with fresh Neurobasal medium (without BrU).
4. Fix the nerve fibers with fresh, cold 4% paraformaldehyde for 20 minutes at 4°C.
5. Wash the samples with PBS (3 x 5’).
6. Incubate with blocking buffer (PBS + 5% normal goat serum) for 45 min at 37°C.
7. Wash the samples with PBS (3 x 5’).
8. Incubate the samples with monoclonal antibody anti-BrdU (concentration 1:200) for 2 hours at 37°C.
9. Wash the samples with PBS (5 x 5‘).
10. Incubate the samples with secondary Alexa-488 antibody for 1 hour at 37°C.
11. Wash samples 5 x 5’ with PBS and mount in glass slides using mounting medium.
12. Visualize samples with confocal fluorescence microscopy.

*Protocol for surface imaging of intact, basal lamina–disrupted and neuropathic nerve fibers.*

1. Immediately after nerve fibre teasing, fix the nerve fibers with fresh, cold 4% paraformaldehyde for 20 minutes at 4°C. Note: For basal lamina disruption experiments, incubate the nerve fibers for 20 minutes at 37°C in Neurobasal medium containing 0.05 % (w/v) of CLSPA collagenase. Then fix the samples with fresh, cold 4% paraformaldehyde for 20 minutes.
2. Wash the samples with PBS (3 x 5’) to remove excess of paraformaldehyde and the remaining digested collagens.
3. Bring the samples to the AFM and image isolated nerve fibers contained in PBS using contact mode. Topographical images of wild-type, basal lamina–digested, and neuropathic *Pmp22-/-* nerve fibers are represented in Figure 5 A, 5 B and 5 C, respectively.

**Supplementary figure 1. Evaluation of nerve fiber structural integrity and damage.** The increase in permeability of 70kDa FITC-Dextran into the nerve fibers is used as marker for sample integrity during AFM-confocal measurements. The presence of dextran (green) inside the axon is imaged by confocal microscopy. The free-labelled myelin and AFM tip are visualized in grey.

**Supplementary video**

**Simultaneous AFM-confocal setup**. Confocal z-stacks showing teased myelinated nerve fibers stained with a myelin marker (red) and immersed in Neurobasal media containing FITC-dextran (green). The latter helps to visualize the non-fluorescent cantilever (triangular shape) and the AFM tip.
