# Supplementary figures and images for: Nano-scale Biophysical and Structural Investigations on Intact and Neuropathic Nerve Fibers by Simultaneous Combination of Atomic Force and Confocal Microscopy
Source: Front Mol Neurosci. 2017 Aug 30;10:277. doi: 10.3389/fnmol.2017.00277 (PMC5582161; doi:10.3389/fnmol.2017.00277)

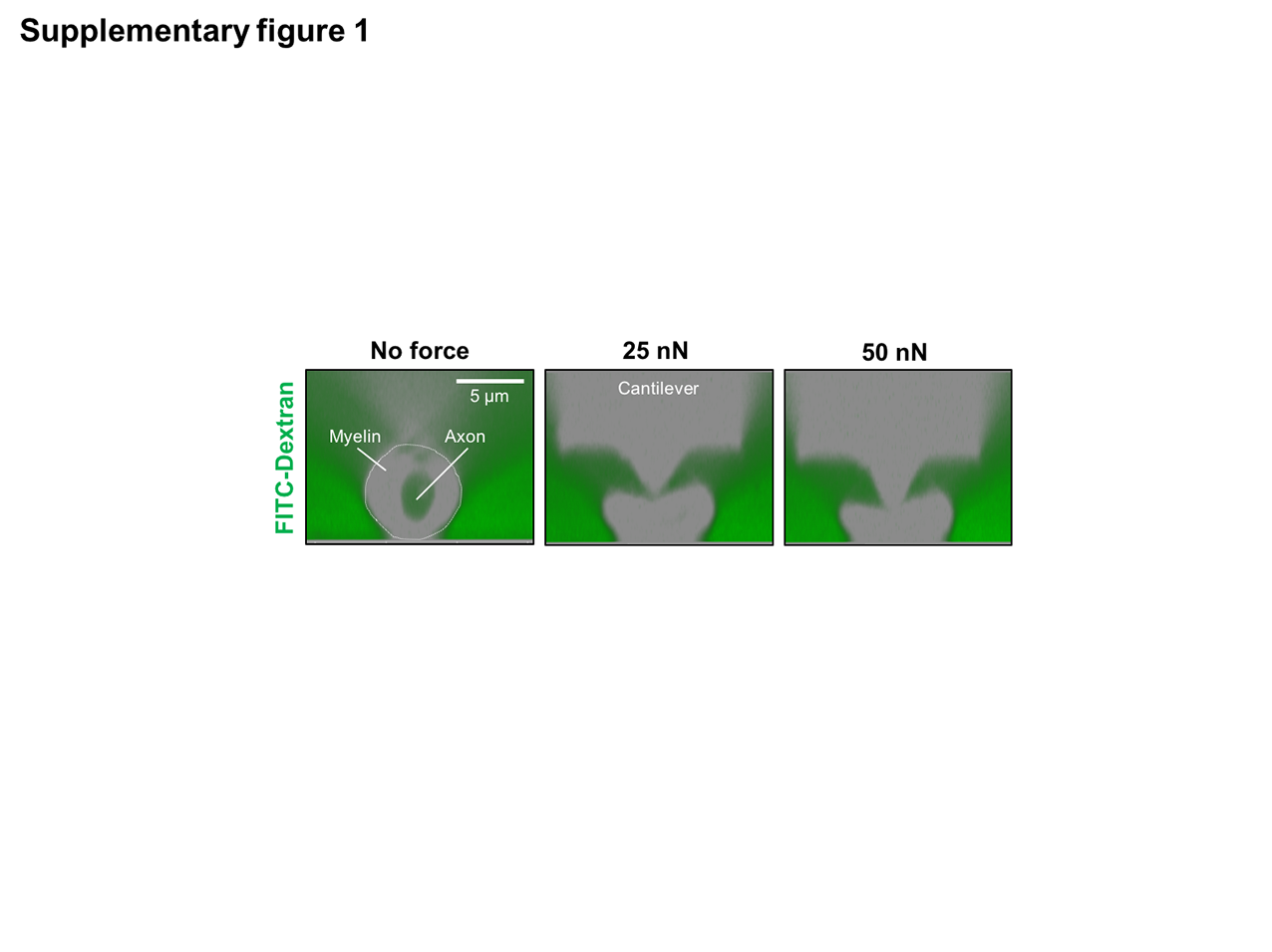

Supplement: Supplementary file 3 [file Image1.TIF]
